# Decoding the impact of gut microbiota on heart failure

**DOI:** 10.1016/j.gendis.2025.101592

**Published:** 2025-03-06

**Authors:** Shuhong Zhao, Lingxuan Dan, Rong Huang, Zhuoyu Shen, Dan Huang, Pan Wu, Zhenguo Ma

**Affiliations:** a Department of Cardiology, Renmin Hospital of Wuhan University, Wuhan, Hubei 430060, China; b Hubei Key Laboratory of Metabolic and Chronic Diseases, Wuhan, Hubei 430060, China; c Department of Adult Internal Medicine, Maternal and Child Health Hospital of Hubei Province, Wuhan, Hubei 430070, China; d Women and Children's Hospital of Hubei Province, Wuhan, Hubei 430070, China

**Keywords:** Gut microbiota, Gut-heart axis, Heart failure, Substance metabolism, Therapeutic modulation

## Abstract

Decreased cardiac output in heart failure leads to intestinal ischemia and increased permeability. Substantial changes occur in the gut microbiota, characterized by a decline in beneficial bacteria and an overgrowth of potentially harmful bacteria. The gut microbiota is intricately linked to prevalent risk factors for heart failure, including hypertension, diabetes, obesity, and renal insufficiency. Furthermore, imbalanced microbiota-derived metabolites enter the bloodstream and may contribute to the progression of heart failure. Ongoing research explores gut microbiota manipulation to alleviate heart failure with probiotics, targeted antibiotics, fecal microbiota transplantation, and dietary adjustments. This review summarizes how gut microbiota participates in heart failure and highlights the emerging promise of modulating gut dysbiosis as a therapeutic approach for managing heart failure.

## Introduction

Cardiovascular diseases (CVDs) consistently rank as the leading cause of global mortality, significantly impacting both health and quality of life. However, the incidence of CVDs is currently increasing year by year.^1^ Heart failure (HF) occurs in the end stage of various CVDs, such as hypertension, myocardial infarction, and myocarditis. It is characterized by cardiac remodeling, which involves various structural and functional changes in the myocardium that develop in response to chronic stress or injury to the heart.^2^ These changes help maintain heart function to some extent. However, in the long term, they tend to accelerate the progression of CVDs and ultimately lead to HF.^3^ Therefore, cardiac remodeling has remained a focal point of our attention and investigation.^4^, ^5^, ^6^, ^7^, ^8^, ^9^, ^10^, ^11^ Despite substantial advancements in the medication for HF, mortality rates from CVDs continue to be disturbingly high. Therefore, developing targeted therapies is of great importance for the prevention and treatment of CVDs.

Recently, the role of the gut microbiota in HF has received extensive attention. In HF patients, substantial changes occur in the gut microbiota, characterized by a decline in beneficial bacteria and an overgrowth of potentially harmful bacteria.^12^ These changes indicate that gut dysbiosis plays an important role in the development of HF, and therapy targeting the gut microbiota may become a new treatment approach. Furthermore, increasing evidence suggests that microbiota-derived metabolites, such as trimethylamine N-oxide (TMAO), bile acids (BAs), short-chain fatty acids (SCFAs), and amino acids (AAs), may influence the development of myocardial remodeling.^13^, ^14^, ^15^, ^16^, ^17^, ^18^ Modulating the composition of the gut microbiota appears to help ameliorate myocardial fibrosis and delay the development of HF.

In the past few years, there has been an explosion of reports regarding gut microbiota, and many excellent review articles have summarized the interactions between the gut microbiota and various organs in the body.^19^, ^20^, ^21^, ^22^, ^23^, ^24^ Here, we focus on the role of gut microbiota in HF and its significance in cardiac remodeling. Although emerging evidence suggests that gut dysbiosis significantly influences the progression of HF, the specific mechanisms remain unclear. Given that HF is a multifactorial chronic condition influenced by numerous risk factors, it is critical to understand how these factors interact with gut dysbiosis in the context of HF. This review creatively explores how gut microbiota-derived metabolites influence HF and associated risk factors via molecular and cellular pathways. Furthermore, we discuss the potential advantages and challenges of novel therapeutic approaches targeting the gut microbiota, aiming to bridge the knowledge gap between gut health and HF, thereby laying a foundation for future research and clinical advancements.

## Composition and function of the gut microbiota

### Composition of the gut microbiota

The gut microbiota is the collection of microorganisms that reside in the digestive system.^25^ The human gut microbiota comprises 10^13^ to 10^14^ microorganisms, and their collective genome, known as the “microbiome”, contains at least 100 times more genes than the human genome.^26^ The intestinal tract harbors several major bacterial groups classified into the *phyla Bacteroidetes*, *Firmicutes*, *Actinobacteria*, and *Proteobacteria*.^27^

### Function of the gut microbiota

Currently, extensive research has elucidated the roles of specific gut microbiota species in a broad array of physiological activities (Table 1).

**Table 1:** The function of the main gut microbiota in the body.

Gut microbiota	Host site	Functions	Reference
*Lactobacillus*	Gastrointestinal tract, oral cavity, vagina, skin, breast	Increases barrier function	28
		Alleviates colitis	29
		Upregulates hepatic antioxidant enzymes and ruminal barrier function	30
		Ameliorates colorectal tumorigenesis	31
		Improves bone loss	32
		Improves insulin resistance	33
*Bifidobacterium*	Colons of newborns and infants, large intestines of adults, oral cavity	Improves cognitive function and mood	34
		Relieves gut dysmotility-related disorders	35
		Prevents colorectal cancer	36
		Enhances host antiviral response	37
		Alleviates colitis	38
*Faecalibacterium prausnitzii*	Terminal ileum, ileocecal region	Suppresses colonic inflammation	39
		Improves the epithelial barrier integrity	40,41
*Clostridium butyricum*	Colon	Alleviates intestinal inflammation	42,43
		Protects intestinal barrier function	44,45
		Promotes bone development	46
*Akkermansia muciniphila*	The mucous layer of the colon	Alleviates intestinal inflammation	47
		Promotes epithelial development	48

In a nutshell, the gut microbiota has two main functions: participation in substance metabolism and regulation of intestinal barrier function. Gut microbiota can degrade carbohydrates, monosaccharides, fats, proteins, and peptides. Specific bacteria possess the capability to degrade cellulose, converting it into SCFAs such as acetic, propionic, and butyric acids. Notably, these compounds serve as the primary energy source for colonic epithelial cells. Furthermore, SCFAs play a critical role in regulating immune responses and modulating inflammation.^49^^,^^50^ In addition, the gut microbiota impacts the synthesis, metabolism, and transportation of fat-soluble vitamins at various levels. Moreover, the synthesis of BAs and AAs is closely associated with the gut microbiota.^51^^,^^52^

Gut microbiota closely interacts with intestinal epithelial cells to reduce the infiltration of harmful substances by producing adhesion proteins and polysaccharides, which strengthen the integrity of the mucosal barrier. Additionally, by competing for nutrients and attachment sites, gut microbiota reduces the colonization of pathogenic bacteria.^53^^,^^54^ It is generally accepted that regulating gut microbiota can improve intestinal barrier function. For example, the probiotic strain of *Lactobacillus plantarum HNU082 (Lp082)* enhanced the chemical barrier through decreased levels of intercellular cell adhesion molecule-1 (ICAM-1) and vascular cell adhesion molecule-1 (VCAM-1). In addition, *Lp082* improved the mechanical barrier by increasing the levels of zonula occludens-1 and zonula occludens-2 while decreasing the levels of claudin-1 and claudin-2.^55^ Certain strains of enteric bacteria can also degrade and eliminate harmful substances, such as toxins and pathogenic factors.

### Relationship between gut microbiota and host health

**Figure 1:**
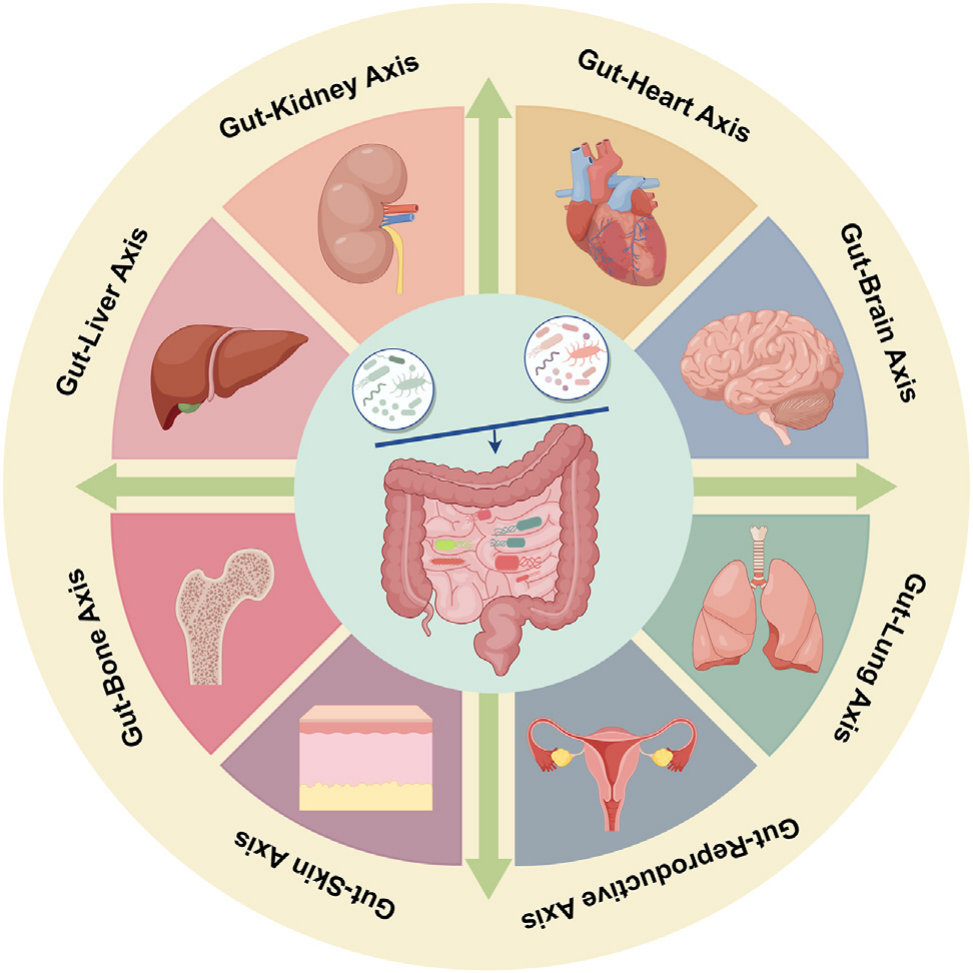
A bidirectional communication system exists between the gut and multiple organs throughout the body. The gut microbiota and their metabolic products engage in interactions with other host organs, creating what is known as cross-talk, which constitutes the host-microbial immune-metabolic axes. Currently, eight axes have been extensively researched and acknowledged: gut-heart axis, gut-brain axis, gut-lung axis, gut-liver axis, gut-kidney axis, gut-skin axis, gut-bone axis, and gut-reproductive axis.

Based on the above functions, strong associations exist between gut microbiota and human health. Currently, gut microbiota is considered to have bidirectional communication with multiple organs of the body, thus affecting the progression of a variety of diseases (Fig. 1).

The gut dysbiosis can lead to a variety of diseases. Conversely, treatments targeting gut microbiota can reverse these conditions. For example, gut dysbiosis plays a pivotal role as a direct instigator of inflammatory bowel disease.^56^ From this point of view, fecal microbiota transplantation (FMT) has become a common treatment for inflammatory bowel disease, benefiting many patients.^57^ Similarly, the disruption of the intestinal barrier is observed in patients with liver cirrhosis, and this disruption forms a vicious cycle that exacerbates disease progression. Many studies have confirmed the role of regulating microbial structure in the treatment of liver cirrhosis. Currently, FMT is gradually being translated into clinical practice to improve the outcome of liver cirrhosis.^58^, ^59^, ^60^ Moreover, gut microbiota has been implicated in the pathogenesis of various autoimmune diseases, including rheumatoid arthritis, systemic lupus erythematosus, and type 1 diabetes mellitus, as well as neuropsychiatric disorders like depression and Alzheimer's disease. Furthermore, a lot of substances from the intestine, such as inflammatory mediators, hormones, and metabolites, can reach the brain and thereby influence brain function.^61^ Intriguingly, gut microbiota can also modulate the synthesis and metabolism of neurotransmitters, including γ-aminobutyric acid, dopamine, and serotonin. These neurotransmitters play crucial regulatory roles in the brain, being intimately associated with emotional states, memory, and cognitive processes.^62^^,^^63^

Last but not least, the connection between the gut microbiota and the heart is equally intimate. HF is associated with chronic low-grade inflammation, a condition that can be exacerbated or reversed by changes in the gut microbiota.^33^^,^^64^ Consistently, clinical evidence suggests that the use of certain probiotics can reduce inflammation in HF patients.^65^ Moreover, gut dysbiosis shares common risk factors with HF. Multiple animal studies have demonstrated that modulating gut microbiota can improve cardiac function and survival rates in HF models.^33^^,^^66^^,^^67^

Indeed, given its vast impact, the gut microbiota is often referred to as the second genome of the human body. Gut dysbiosis affects the overall state of the body beyond simple intestinal diseases. It can influence various physiological and pathological processes, including immune function, metabolic regulation, and even neurological function. Thus, investigating the multifaceted roles of the gut microbiota in both health and disease is of paramount importance.

## The impact of gut microbiota on HF

### Gut microbiota and risk factors of CVDs

With the increasing prevalence of poor lifestyle choices, there is a corresponding rise in the prevalence of individual risk factors for HF, including hypertension, diabetes, and obesity. Gut microbiota can influence metabolism, immune system, and inflammatory response, all of which can contribute to the development of HF and high-risk factors for CVDs (Table 2). Furthermore, several additional factors are associated with CVDs, which may stem from or exacerbate gut dysbiosis. These factors include, but are not limited to, smoking, dietary patterns, environmental pollutants, and renal insufficiency.^68^, ^69^, ^70^, ^71^ Each of these elements independently and cumulatively contributes to CVDs, highlighting the complexity of the interplay between gut microbiota and cardiovascular health.

**Table 2:** The role of gut microbiota in the risk factors of CVDs.

Risk factors	Model	Observations	Interventions and effects	Reference
Hypertension	Chronic Ang II infusion	Decreased microbial richness Increased ratio of *Firmicutes* to *Bacteroides*	Minocycline: lowers blood pressure and restores the ecological balance of gut microbiota	72
	Angiotensin II infusion + apolipoprotein E-deficient	Myocardial hypertrophy, myocardial fibrosis, and vascular dysfunction	Propionate: attenuates hypertension	73
	Spontaneously hypertension	Increased ratio of *Firmicutes* to *Bacteroides* Lower acetate- and higher lactate-producing bacteria	Losartan: alleviates intestinal ecological imbalance and intestinal sympathetic drive	74
	High-salt diet	Increased intestinal inflammation Increased *Firmicutes, Proteobacteria*, and *Prevotella*	Fecal microbiota transplantation from high-salt diet mouse to germ-free mouse: make intestinal inflammation and hypertension prone to occur	75
	Angiotensin II infusion	Vascular dysfunction and hypertension	Germ-free mice: relieves cardiac inflammation, fibrosis and systolic dysfunction	76
Diabetes	HFD + streptozotocin-induced dilated cardiomyopathy	Increased *Clostridiales* and *Lachnospiraceae* increased intestinal permeability	Pyridostigmine: improves the intestinal barrier, increases ATP production, and attenuates cardiac dysfunction, hypertrophy, and fibrosis	77
	HFD-induced obesity and diabetes	Decreased abundance of *Blautia* Increased abundance of *Faecalibacterium*, *Butyricicoccus*, and *Megasphaera*	*Blautia wexlerae*: alleviates T2DM via altering metabolism and exhibiting anti-inflammatory effects	78
	HFD-induced obesity	Increased concentrations of secondary BAs and SCFAs	Vitamin K2: improves impaired glycemic homeostasis and insulin sensitivity in T2DM through the gut microbiota and fecal metabolites	79
	Streptozotocin-induced T2DM	Lower community richness and evenness	Papeiflorin: ameliorates cardiac dysfunction and myocardial injury via altering the structure and composition of the gut microbiota community	80
	HFD-induced obesity and diabetes	Increased *Bacteroides fragilis* and decreased GUDCA Glucose intolerance and insulin resistance	Metformin: improves metabolic dysfunction part through a *B. fragilis*–GUDCA–intestinal FXR axis	81
	db/db mice	High blood glucose levels and insulin resistance Gut microbiota perturbations and disruption of gut barrier function	Cholinergic drugs: mitigates diabetic cardiac injury by augmenting the quantity of anti-inflammatory bacteria while diminishing the quantity of pro-inflammatory bacteria through the LPS-1-mediated ERK/Egr-1 pathway	82
Obesity	HF + intermittent hypoxia-induced Obstructive sleep apnea	Changed gut microbiota Cardiac dysfunction	*Lactobacillus rhamnosus* GG: improves cardiac dysfunction and reduces cardiac remodeling and inflammation in obstructive sleep apnea mice via the activation of the Nrf2 pathway	83
	HFD-induced obesity	Decreased capacity to metabolize ethanolamine	Probiotic: restores the activity of ethanolamine-β-lactamase and reduces intestinal permeability and inflammation	84
	HFD-induced obesity	Glucose intolerance, insulin resistance, and hyperinsulinemia Impaired gut barrier function	Spermidine: decreases metabolic endotoxemia and improves intestinal barrier integrity through autophagy pathway and TLR4-mediated microbial signal transduction	85
	HFD-induced obesity	Decreased fermentation of SCFAs and transformation of BAs Increased metabolism of BCAAs	Co-housing Ob and Ln mice: prevents weight gain and obesity-related metabolic phenotypes in obese mice	86

Note: HFD, high-fat diet; GUDCA, glycochenodeoxycholic acid; BCAAs, branched-chain amino acids; BAs, bile acids; SCFAs, short-chain fatty acids; T2DM, type 2 diabetes mellitus.

### Gut microbiota and HF

HF is the ultimate common process caused by various initial heart injuries. It is triggered by an imbalance in compensatory mechanisms and pathogenic processes. Numerous clinical and basic studies have consistently demonstrated the critical role that gut microbiota plays in myocardial fibrosis and HF. Research has shown that transverse aortic constriction surgery administered to germ-free mice often results in more severe cardiac hypertrophy and cardiac dysfunction compared with the control group. Conversely, supplementation of acetate and propionate can improve heart function, reduce fibrosis, and reverse extracellular matrix dysfunction.^87^ In the transverse aortic constriction-induced cardiac remodeling model, 16S rRNA sequencing of fecal samples revealed a reduction in bacteria-producing tryptophan and SCFAs in wild-type mice.^14^ Mice treated with antibiotics demonstrated a notable dose-dependent increase in mortality compared with the control when subjected to myocardial infarction. High-performance liquid chromatography analysis showed a decrease in SCFAs after antibiotic treatment. Following fecal reconstruction and nutritional SCFA supplementation, the mice's survival rate and physiological state were significantly improved.^18^ Facts have shown that both reactive fibrosis and reparative fibrosis are affected by gut microbiota extensively. It was discovered that the gut microbiota of HF patients was primarily distinguished by a decline in both diversity and composition. In particular, there was an increase in *Proteobacteria* and *Candida* and a decrease in the number of taxa in the *Firmicutes*.^88^^,^^89^ *Lactobacillus* is one of the most extensively studied genera in the gut microbiota. Research has shown that *Lactiplantibacillus plantarum P470* has the potential to improve the gut microbiota in the feces of patients with coronary heart disease.^90^ On the contrary, *Proteobacteria* include many potential pathogens, such as *Escherichia coli* and *Salmonella*. An increase in pathogenic bacteria is associated with heightened inflammation and infection risk in HF patients.^16^

### The gut microbiota acts directly on the failing heart

The structure and function of the gastrointestinal tract in patients with HF are altered.^91^^,^^92^ When HF occurs, the decrease in cardiac output and the activation of the neuro-humoral system stimulate the redistribution of systemic blood, leading to insufficient perfusion of the gastrointestinal tract.^93^ Structurally, intestinal wall thickening and intestinal edema are often observed. Functionally, intestinal permeability increases and intestinal barrier function is impaired.^92^ Accompanied by the increase in intestinal inflammation and permeability, gut pathogenic microbiota, and their toxins can translocate from the intestinal tract to the bloodstream, causing systemic chronic inflammation and contributing to chronic HF.^89^^,^^94^ Elevated levels of inflammatory factors are frequently associated with worse prognosis and more severe clinical symptoms in HF patients. Evidence suggests that these inflammatory mediators are linked to fibrosis, apoptosis, and hypertrophy of cardiomyocytes.^95^ Additionally, a significant increase in bacterial concentration and adhesion in the intestinal mucosal biofilm has been observed in patients with HF.^91^^,^^93^ Mamic et al conducted a nationwide study of hospitalized patients and found that those with HF had higher rates of sepsis, pneumonia, and urinary tract infections, indirectly confirming the high inflammatory state of patients with HF.^96^ A higher infection rate of *Clostridium difficile* is observed in hospitalized HF patients. Therefore, the disorder of gut microbiota can directly affect the body, leading to the progression and deterioration of HF.

### The gut microbiota acts indirectly on the failing heart through its metabolites

**Figure 2:**
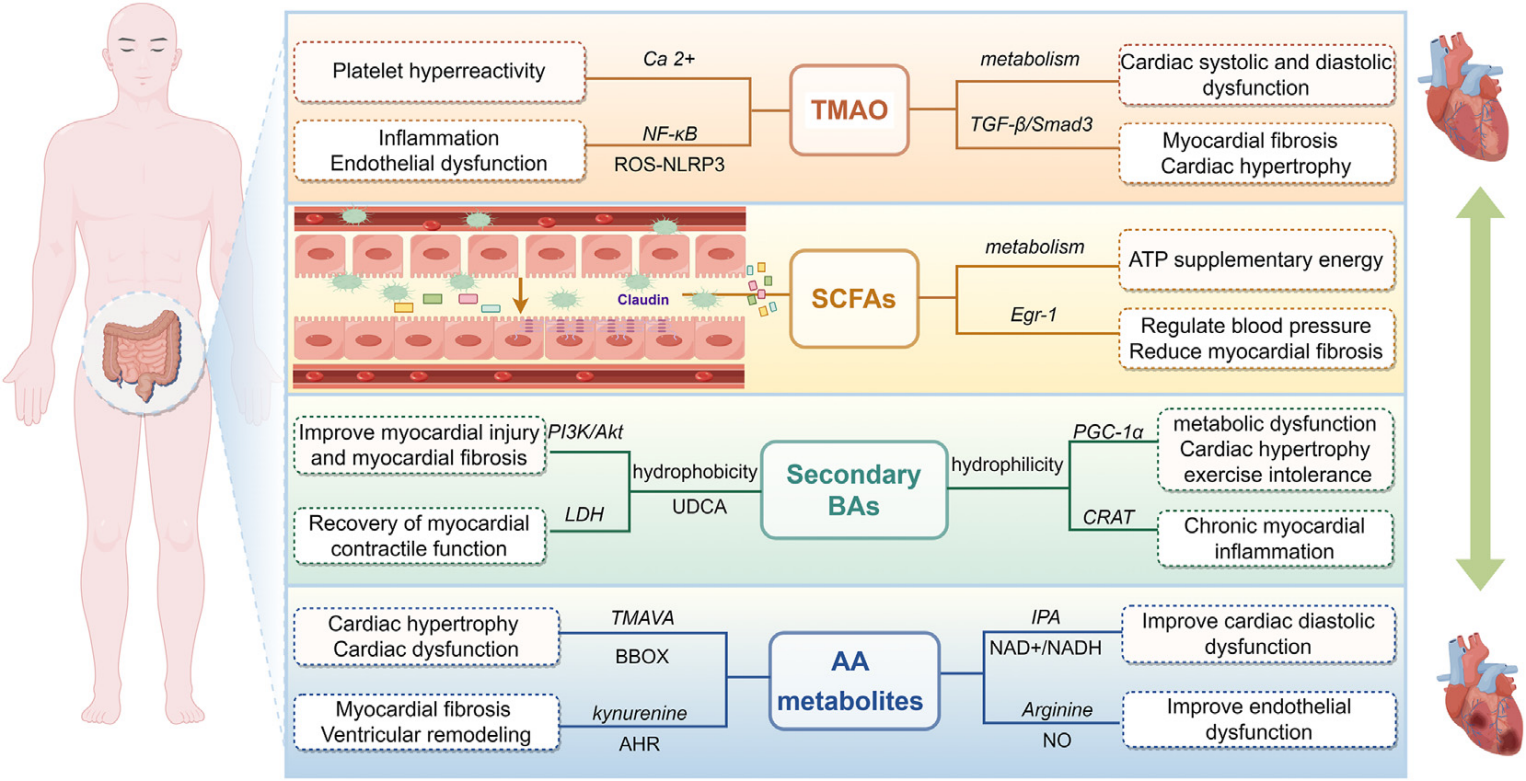
Mechanisms of dietary metabolites generated by the gut microbiota in heart failure. Through the processes of intestinal motility and the metabolic activities of the gut microbiota, substances such as TMAO, SCFAs, BAs, and AAs are synthesized in the intestine. In patients with heart failure, an imbalance in the metabolism of these substances serves as a critical indirect driver that either triggers or worsens the condition. TMAO, trimethylamine N-oxide; BAs, bile acids; SCFAs, short-chain fatty acids; AAs, amino acids.

The metabolic substrates for gut microbiota primarily originate from food that the host cannot efficiently digest, as well as mucus secreted by intestinal epithelial cells. Following the activity of gut microbiota, a multitude of metabolites are produced, including both detrimental and beneficial compounds such as lipopolysaccharides, peptidoglycans, trimethylamine, BAs, and SCFAs.^12^ Numerous studies have demonstrated that the gut microbiota-derived metabolites can significantly influence systemic health. (Fig. 2).

### TMAO

TMAO is a metabolite derived from dietary choline and has been shown to promote the development of atherosclerosis through various mechanisms. On one hand, TMAO can stimulate inflammation and endothelial dysfunction by activating the reactive oxygen species/thioredoxin-interacting protein (TXNIP)/NOD-like receptor family pyrin domain-containing 3 (NLRP3) inflammasome pathway.^97^ On the other hand, TMAO exhibits pro-thrombotic potential *in vivo*.^98^ Supplementation with dietary choline and TMAO accelerates atherosclerotic plaque formation in mice.^99^ In a prospective study conducted by Tang et al, fasting TMAO levels were significantly elevated in patients with HF compared with those without HF, thereby suggesting an increased risk of long-term mortality.^100^ These findings have been corroborated in animal research as well. In a mouse model of doxorubicin-induced cardiac fibrosis, a TMAO-enriched diet exacerbated cardiac dysfunction and myocardial fibrosis. TMAO promoted collagen deposition, fibrosis, and inflammatory factor expression in a dose-dependent manner through the activation of the transforming growth factor beta1 (TGF-β1)/Smad3 signaling pathway.^101^ 3,3-Dimethyl-1-butanol has been shown to mitigate TMAO levels in HF mice by inhibiting the p65 nuclear factor kappa B (NF-κB) signaling pathway and the TGF-β1/Smad3 signaling cascade, thereby ameliorating the adverse cardiac remodeling driven by TMAO.^102^ The research by Organ et al directly substantiated the detrimental impact of TMAO on the heart. In comparison with a standard diet, a TMAO-enriched diet was found to notably exacerbate myocardial hypertrophy, myocardial fibrosis, and the severity of HF in mice following transverse aortic constriction.^103^ Similarly, neonatal rat cardiomyocytes exposed to TMAO *in vitro* exhibited increased cell size and up-regulated the expression of hypertrophic markers such as atrial natriuretic peptide (ANP) and β-myosin heavy chain (β-MHC).^104^ The increased concentration of TMAO in plasma and heart tissue can directly inhibit pyruvate and fatty acid oxidation. The disorder in cardiac energy metabolism leads to both systolic and diastolic dysfunction in cardiomyocytes.^105^ Moreover, TMAO has been shown to stimulate the elevation of inflammatory cytokines and the accumulation of reactive oxygen species within the body, leading to an inflammatory response and direct injury to cardiomyocytes.^106^ Consequently, TMAO has been substantiated as a promising cardiovascular risk biomarker and holds potential as a therapeutic target in HF management.

### SCFAs

SCFAs are produced through the anaerobic fermentation of dietary fiber by colonic bacteria or yeast.^107^ The most prevalent SCFAs in the body include acetic acid, propionic acid, and butyric acid.^108^ The primary bacterial genera responsible for producing SCFAs include *Bacteroides*, *Bifidobacterium*, *Firmicutes*, *Ruminococcus*, *Lactobacillus*, *Clostridium*, and *Streptococcus*.^107^ Currently, many studies have demonstrated that SCFAs can enhance intestinal barrier integrity, modulate the immune system, regulate inflammatory responses, improve glucose and lipid metabolism, and influence blood pressure.^109^, ^110^, ^111^, ^112^ As previously discussed, the levels of SCFAs are significantly reduced in patients with HF compared with healthy controls, while exogenous supplementation has been shown to mitigate the progression of HF. Specifically, butyrate potentiates the integrity of intestinal epithelial tight junctions by up-regulating tight junction components and stimulating protein recombination. It preserves intestinal barrier function and reduces the ingress of detrimental substances, such as endotoxins, into the systemic circulation. In essence, SCFAs can reduce the risk of systemic inflammation.^113^ Moreover, transcriptome analysis showed that dietary supplements high in fiber and acetate reduced the proportion of *Bacteroides* and *Prevotella*, accompanied by a down-regulation of early growth response 1 (Egr-1) in the heart and kidney, which significantly reduced systolic and diastolic blood pressure, cardiac fibrosis, and left ventricular hypertrophy.^114^ SCFAs also compensate for the impaired oxidation capacity of long-chain fatty acids in the hypertrophic heart, serving as an alternative substrate for ATP production through oxidation.^115^ Therefore, it can be identified that SCFAs hold considerable significance in the prevention and management of HF.

### BAs

BAs are recognized as emulsifiers of fats and fat-soluble vitamins. Primary BAs are synthesized from cholesterol in the liver, while secondary BAs are produced by bacteria residing in the intestinal tract.

Several studies have demonstrated that the total concentration and composition ratio of BAs significantly altered in patients with HF. A prospective clinical investigation found that plasma BA levels in HF patients were notably lower than those in healthy controls. This decline was accompanied by an increase in the ratio of secondary to primary BAs. Through univariate analysis, it has been demonstrated that the altered pattern of BAs correlates with a reduced overall survival rate in patients with HF.^116^ In a BA overload model, pronounced cardiac hypertrophy and exercise intolerance were observed. Further research has revealed that elevated serum BA levels can suppress the expression of peroxisome proliferator-activated receptor gamma coactivator 1-alpha (PGC-1α), thereby inhibiting fatty acid oxidation both *in vivo* and *in vitro*, which ultimately leads to biliary and cardiac metabolic dysfunction.^117^ In dysfunctional cardiomyocytes, the depletion of carnitine acetyltransferase promotes cholesterol utilization for BA synthesis. The intracellular accumulation of BAs or their intermediate metabolites results in the release of mitochondrial DNA into the cytosol, which subsequently initiates a type I interferon (IFN–I) response and activates absent in melanoma 2 (AIM2) inflammasomes. This cascade ultimately contributes to the progression of chronic myocardial inflammation and HF.^118^

BAs can be classified into hydrophobic BAs and hydrophilic BAs based on their distinct structures. The former category is known for inducing cytotoxicity and apoptosis, and the latter exhibits a reduced cytotoxic effect. Ursodeoxycholic acid stands out as the most hydrophilic of the cholic acids. Initially used in the treatment of chronic liver diseases, ursodeoxycholic acid can also decrease lactate dehydrogenase release and enhance the recovery of cardiac systolic function in the ischemia/reperfusion model.^119^ Moreover, ursodeoxycholic acid ameliorates myocardial infarction and inhibits mitochondrial permeability transition pore opening in a phosphoinositide 3-kinase (PI3K)/protein kinase B (PKB/Akt)-dependent pathway.^120^ Ursodeoxycholic acid can also exert inhibitory effects on apoptosis and reduce myocardial hypertrophy and fibrosis.^121^ Therefore, BAs significantly influence the progression and management of HF, positioning them as a potential therapeutic target for intervention.

### AAs

Alterations in plasma AAs and their derivatives are detectable in patients with HF and have been deemed as early indicators of CVDs.^122^ Zhao et al identified a strong association between increased levels of N-trimethyl-5-aminopentanoic acid and the risks of heart-related death and transplantation. This acid can interfere with cardiac energy metabolism via the γ-butyrobetaine hydroxylase (BBOX) pathway. It inhibits fatty acid oxidation and leads to the accumulation of lipids, thus exacerbating cardiac hypertrophy and dysfunction.^123^ Likewise, through evidence supported by single-nucleus RNA sequencing, it has been established that certain molecules can activate aromatic hydrocarbon receptors and contribute to ventricular remodeling.^124^ During the progression of HF, impairments in AA metabolism can result in inadequate energy supply to the myocardium, exacerbating damage to cardiomyocytes. However, the role of AAs and their metabolites in HF has yielded varying outcomes across different studies. In the pressure overload model, replacing dietary AAs has been shown to improve systemic glucose metabolism and rebalance metabolic substrate utilization in HF by enhancing mitochondrial fuel oxidation efficiency.^125^ Furthermore, Wang et al have demonstrated that FMT can restore cardiac levels of nicotinamide and sirtuin 3 (SIRT3). This restoration subsequently reduces metabolic remodeling and inflammation, thereby mediating a protective effect against diastolic dysfunction in the heart.^126^

The diverse roles played by AAs and their metabolites in CVDs can be attributed to the body's energy intake status. In instances of surplus energy, excessive consumption of AAs might elevate the risk of HF, whereas in conditions characterized by energy insufficiency, dietary supplementation with AAs could promote health.

## Immune regulation induced by gut microbiota

Gut microbiota can produce a variety of immunomodulatory factors that directly or indirectly regulate the activity of immune cells, such as dendritic cells and CD4^+^ T cells.^127^^,^^128^ Gut microbiota can modulate the inflammatory response of the immune system by activating signaling pathways such as Toll-like receptors (TLRs), NF-κB, and mitogen-activated protein kinases (MAPKs).^127^ SCFAs also have important effects on immune cell activity and function. The effects of butyrate on immune cells are mainly mediated in three ways: direct activation of surface G-protein-coupled receptors (GPCRs), activation of peroxisome proliferator-activated receptor gamma (PPARγ), and inhibition of butyrate-sensitive histone deacetylase (HDAC) isoforms.

An imbalance in the gut microbiota can lead to excessive growth of pathogenic microorganisms, resulting in the release of large amounts of endotoxins and inflammatory agents. This fosters chronic low-grade systemic inflammation, which increases susceptibility to various illnesses, including CVDs, autoimmune disorders, and certain metabolic conditions, such as atherosclerosis. Concurrently, a reduction in beneficial microbiota weakens resistance against infections, increasing the likelihood of pathogen invasion. For example, rheumatic heart disease, which is caused by an autoimmune response to *group A streptococcal* infection, has been shown to be closely related to changes in gut microbiota.^129^ Experiments in myocardial infarction models showed improvements after FMT, monocyte transfer, or SCFA dietary supplementation and highlighted the relevance of immune modulation in treating CVDs.^18^

## Novel therapeutic interventions in HF

### Probiotic bacteria

The decline in the number and relative abundance of beneficial gut microbiota in HF patients can exacerbate systemic inflammation and accelerate the progression of HF. Probiotics can stimulate the proliferation of specific microbial populations and enhance the balance of the gut microbiota. Components intrinsic to probiotics, such as peptidoglycan and lipoteichoic acid, can serve as antigens and exert both direct and indirect immune-stimulatory effects, thereby enhancing overall immunity.^130^ Previous investigations have shown that administering *Lactobacillus* and probiotics before myocardial infarction in mice shifted the balance of SCFAs towards propionate and conferred cardioprotective benefits.^18^ Another study conducted by Gan et al also observed a protective effect of *Lactobacillus rhamnosus* on the heart following myocardial infarction.^131^ In a randomized controlled clinical trial, consuming probiotic yogurt over 10 weeks effectively improved the inflammatory status in patients with chronic HF.^132^^,^^133^ Furthermore, probiotics can facilitate nutrient digestion and absorption, indirectly influencing cardiovascular metabolism. Notably, despite probiotics generally having an excellent safety profile, a lack of regulation may raise the risk of unintended translocation of probiotics into the bloodstream, potentially leading to sepsis. Therefore, careful consideration and appropriate regulation are essential when administering probiotics.

### Diet

CVDs are intrinsically linked to metabolism. Hence, the importance of adopting healthy dietary habits is quite evident. Both in humans and mice, high salt intake has been associated with an increase in certain bacteria, such as *Streptococcus*, *Proteus*, and *Prevotella*, which predisposes mice to vascular inflammation and hypertension.^75^ The dietary approach to stop hypertension (DASH) diet, recommended by the American Heart Association guidelines, emphasizes a rich intake of fruits, vegetables, whole grains, and lean products while limiting the consumption of sugary foods, beverages, red meat, and saturated fats.^134^ Currently, accumulating evidence supports the benefits of the DASH diet in HF patients. A high-fiber diet has notably been shown to decrease systolic and diastolic blood pressure, cardiac fibrosis, and left ventricular hypertrophy, partly through the down-regulation of Egr-1 in the heart and kidneys.^114^ Diets enriched with fermented foods consistently enhance microbiota diversity and diminish inflammatory biomarkers.^135^ Moreover, the CORDIOPREV study suggests that a Mediterranean diet intervention may effectively reduce the risk of myocardial infarction, revascularization, and ischemic stroke, outperforming a low-fat diet approach.^136^ Beyond monitoring food choices, paying attention to eating patterns is equally significant. Research indicates that intermittent fasting practices, such as alternate-day fasting or time-restricted feeding, may assist in lowering the risk of CVDs.^137^

### FMT

As a novel therapeutic approach, FMT has garnered substantial interest in the realm of HF research in recent years. FMT involves the transfer of a donor's fecal microbial community to a patient, aiming to restore or reconstruct the balance of gut microbiota and thereby enhance the overall health status of the recipient. When applied to HF model organisms, introducing healthy microbiota has been shown to improve cardiac function, attenuate myocardial fibrosis, suppress inflammation, and facilitate the restoration of metabolic homeostasis. FMT has gained widespread acceptance and utilization in patients suffering from recurrent *Clostridioides difficile* infection.^138^ Nevertheless, the application of FMT in HF remains at an exploratory stage. Further clinical studies are essential to rigorously validate its safety and efficacy specifically for HF patients. With the ever-deepening comprehension of the intricate relationship between the gut microbiota and CVDs, FMT holds promise as a potential new strategy for the management and treatment of HF.

### Rational use of antibiotics

Infection, often a principal trigger for acute exacerbations of HF, ranks among the primary reasons for hospitalizations and fatalities in HF patients. Hence, numerous studies have concentrated on the application of antibiotics to manage infections instigated by intestinal pathogens. Doxycycline, for instance, has been shown to mitigate left ventricular remodeling in patients with myocardial infarction.^139^ However, antibiotics pose a dualistic challenge to the gut microbiota. Improper usage of antibiotics, particularly misuse or deviation from recommended indications, doses, and treatment durations, can engender gut microbiota imbalance and deplete the body's normal symbiotic microbial community. Experimental models demonstrated that antibiotic-treated mice exhibited a severe, dose-dependent increase in mortality post-myocardial infarction.^18^ Therefore, the judicious use of antibiotics transcends merely preventing the emergence of drug resistance. It is also crucial for preserving the balance of the gut microbiota, thereby maintaining gut health and overall well-being. Indeed, nonspecific antibiotic interventions might inflict more harm than benefit.

## Conclusion and prospect

The results of clinical and basic studies have revealed that the changes in gut microbiota significantly influence the progression of HF. There is a decrease in beneficial species such as *Lactobacilli* and *Bifidobacteria*, concurrent with an increase in potentially harmful bacteria like *Clostridia* and *Bacteroides*. A compromised intestinal barrier enables bacterial translocation and abnormal production of metabolites, amplifying inflammation and promoting cardiac remodeling. We have discussed the effects of gut dysbiosis on the host's health, particularly the occurrence of HF. However, given that the gut microbiota plays a role in the metabolism of various substances, the relationship between different types of microbiota-derived metabolites and HF remains to be further elucidated. Additionally, further research should focus on identifying specific molecules and their pathways.

In addition to descriptive investigations, we have also attempted to reveal the cellular and molecular mechanisms of gut microbiota causing HF. As a result, in terms of therapeutic strategy development, HF can be alleviated by regulating gut dysbiosis, and new therapeutic drugs can be developed based on microbiota-derived metabolites. Probiotics, prebiotics, and FMT have demonstrated potential in both experimental studies and clinical scenarios to enhance the prognosis of HF. We have summarized the therapeutic effects and accessibility of the above interventions. However, many issues still need to be further addressed in current clinical treatment strategies. On one hand, longitudinal studies are needed to track the long-term effects of microbiota changes on cardiovascular health due to the gut's dynamism. On the other hand, more extensive and rigorous randomized controlled trials are required to validate their therapeutic benefits and long-term safety. Moreover, the gut microbiota plays an active role in drug metabolism, affecting HF patients' response to standard treatments. Therefore, studying the interaction between the microbiota and drug metabolism is the key to optimizing regimens, enhancing drug effectiveness, and reducing side effects.

In summary, the relationship between gut microbiota and HF opens a promising research field, shedding new light on disease etiology and progression and potentially introducing novel therapeutic approaches. To incorporate gut microbiota regulation into HF management, ongoing basic research and prospective clinical trials are essential to deepening our foundational knowledge.

## Outstanding questions

To thoroughly examine the pivotal role of gut microbiota in CVDs and effectively translate these findings into therapies, several key areas require focused research: i) Determine causality: Is gut microbiota disruption a direct cause or a secondary effect in HF? Longitudinal studies and mechanistic experiments are essential to establish causality. ii) Identify metabolites: Are there unknown critical metabolites produced by gut microbiota, and what are their precise impacts on cardiovascular health? iii) Assess therapeutic potential: Can manipulating gut microbiota serve as an effective adjunct therapy for managing HF? iv) Develop personalized interventions: How can individualized gut microbiota intervention strategies be designed for HF patients with different disease etiologies and stages of progression?

## CRediT authorship contribution statement

**Shuhong Zhao:** Writing – original draft, Visualization, Conceptualization. **Lingxuan Dan:** Writing – original draft, Conceptualization. **Rong Huang:** Writing – review & editing. **Zhuoyu Shen:** Writing – review & editing. **Dan Huang:** Writing – review & editing. **Pan Wu:** Writing – review & editing, Conceptualization. **Zhenguo Ma:** Writing – review & editing, Supervision, Conceptualization.

## Funding

This work is supported in part by grants from the 10.13039/501100001809National Natural Science Foundation of China (No. 82070410, 82270248), the Young Top-notch Talent Cultivation Program of Hubei Province (China), the Knowledge Innovation Program of Wuhan-Basic Research (China), and the Fundamental Research Funds for the Central Universities (China) (No. 2042021kf0205). The funders did not have any role in the paper design, data collection, data analysis, interpretation, and writing of the paper.

## Conflict of interests

The authors declared no competing interests.
